# A Multiparameter Pressure–Temperature–Humidity Sensor Based on Mixed Ionic–Electronic Cellulose Aerogels

**DOI:** 10.1002/advs.201802128

**Published:** 2019-02-07

**Authors:** Shaobo Han, Naveed Ul Hassan Alvi, Lars Granlöf, Hjalmar Granberg, Magnus Berggren, Simone Fabiano, Xavier Crispin

**Affiliations:** ^1^ Laboratory of Organic Electronics Department of Science and Technology Linköping University S‐60174 Sweden; ^2^ Papermaking & Packaging RISE Bioeconomy Box 5604 S‐11486 Sweden

**Keywords:** aerogels, ionic–electronic mixed conductors, multiparameter sensors, poly(3,4‐ethylenedioxythiophene) (PEDOT), thermoelectric materials

## Abstract

Pressure (*P*), temperature (*T*), and humidity (*H*) are physical key parameters of great relevance for various applications such as in distributed diagnostics, robotics, electronic skins, functional clothing, and many other Internet‐of‐Things (IoT) solutions. Previous studies on monitoring and recording these three parameters have focused on the integration of three individual single‐parameter sensors into an electronic circuit, also comprising dedicated sense amplifiers, signal processing, and communication interfaces. To limit complexity in, e.g., multifunctional IoT systems, and thus reducing the manufacturing costs of such sensing/communication outposts, it is desirable to achieve one single‐sensor device that simultaneously or consecutively measures *P*–*T*–*H* without cross‐talks in the sensing functionality. Herein, a novel organic mixed ion–electron conducting aerogel is reported, which can sense *P*–*T*–*H* with minimal cross‐talk between the measured parameters. The exclusive read‐out of the three individual parameters is performed electronically in one single device configuration and is enabled by the use of a novel strategy that combines electronic and ionic Seebeck effect along with mixed ion–electron conduction in an elastic aerogel. The findings promise for multipurpose IoT technology with reduced complexity and production costs, features that are highly anticipated in distributed diagnostics, monitoring, safety, and security applications.

## 1 Introduction

Pressure, temperature, and humidity (*P–T–H*) are crucial physical parameters that, to an extent, describe our environment. They are affecting all living organisms and, in particular, are important conditions for human health and wellbeing. Thus, these parameters are constantly forecasted by meteorological stations and routinely selected by individuals and society. In the case of human body, it is important to monitor how the *P–T–H* parameters vary locally, for instance, to record human responses to extreme conditions,^1^ for medical diagnostics,^2^, ^3^, ^4^ and to study the outcome of physical exercising.^5^ Further, from a technological viewpoint, sensing *P–T–H* is beneficial to many applications such as robotics,^6^ electronic skin,^3^, ^7^, ^8^ or smart packaging (e.g., food and drugs).^9^ Sensors are also heavily explored in the area of Internet of Things (IoT) and “Internet of Everything,” a technology area that will provide a huge amount of data dumped into the cloud, that can be utilized in combination with deep‐learning protocols. Such a system will provide a distributed technology for self‐learning, self‐controlling and self‐therapy solutions and for safety, security, and transportation in our future society.

A first and significant approach to multiparameter sensing is to create sensor arrays.^10^, ^11^ Such arrays usually combine several single‐function sensors, which are based on different sensing materials included in a variety of dedicated transducer structures. Each single‐function sensor responds to one specific stimulus that then results in an exclusive read‐out signal. Therefore, a large number of signals from the environment can be detected at the same time without any cross‐talk. The drawback of sensor arrays is that the fabrication of electronic arrays typically requires complex manufacturing processes (many materials and exclusive production steps) and high costs. In many IoT applications, typically one or several Si chips are combined with an array of different sensors and matrices, thus a large number of individual contact pads and connections are needed. This will severally drive up the cost of IoT communication outposts, where each requires physical contacting based on, e.g., flip‐chip mounting or wire‐bonding, thus limiting multifunctional applications to a large extent based on performance‐over‐cost arguments. A second approach is to use a multifunctional material that responds to a number of parameters but provides one single output signal.^12^, ^13^, ^14^ For instance, conductive polyamide fibers have proven to be sensitive to temperature, humidity, and strain.^12^ This approach reduces the fabrication cost but it is not possible to distinguish between the stimuli since they all lead to changes in the same type of output signal. The challenges and limitations of the first and second approaches described above can be overcome by creating multiparameter sensors with a single multifunctional material that transforms each stimulus into different recordable signals^15^ or into one output signal with different “orthogonal” and independent features that can be separately resolved by sense amplification circuits. This third approach combines the advantages of the two strategies; absence of cross‐talk and simple fabrication thanks to a single device approach. Suppression or circumvention of sensor parameter cross‐talk can be reached through various pathways. For instance, the simple steady‐state current–voltage (*I–V*) characteristics of a device can contain systematic variations of threshold, offset, linear slope, inflection points, or polynomial versus exponential evolution upon exposure to stimuli. Further, changes in hysteresis and dynamic characteristics of the fundamental *I–V* characteristics can be used as well, to further broaden the ensemble of orthogonal sensor parameters. If the variation of one particular *I–V* feature can be exclusively coupled to one specific sensing function, a multiparameter sensor can be derived with orthogonal sensing capability. The challenge lies in decoupling the various readable signals in order to extract the values of each environmental parameter at a local scale. A successful example of this approach is to create a pressure–temperature (*P–T*) dual‐parameter sensor using a thermoelectric material mixed with an elastic scaffold.^16^, ^17^ The temperature is here related to the generated thermoelectric voltage caused by the Seebeck effect (voltage offset) and the pressure stimuli, which is measured as the resistance of the sensor (linear slope). For example, we recently reported an aerogel made of cellulose nanofibrils and the conducting polymer poly(3,4‐ethylenedioxythiophene):polystyrene sulfonate (PEDOT:PSS)^16^, ^17^, ^18^, ^19^, ^20^, ^21^, ^22^, ^23^, ^24^, ^25^, ^26^ with fully decoupled *P–T* sensing capability.^17^

The next challenge is to investigate the possibility to “orthogonally” add humidity sensing in addition to pressure and temperature. Humidity can be measured through several sensor strategies such as via protonic conduction‐type, impedance‐type, and capacitive‐type humidity sensors.^27^ It is inspiring to refer to the human skin that is equipped with cutaneous mechanoreceptors and thermoreceptors. Surprisingly, the skin does not possess any humidity receptor, but cutaneous wetness sensation is coming from the kinetic signal from other receptors.^28^ Hence, adding the time domain (dynamics) for one of the signals could enable orthogonal *P–T–H*‐parameter sensing. Recently, it has been demonstrated that measuring thermovoltage versus time in mixed ionic–electronic conductors (MIECs) allows for sensing temperature gradients and humidity.^29^ MIECs can swiftly transport both ions and electrons. Solid MIECs, such as ceramics and conducting polymers, have been studied as electrodes in fuel cells,^30^ supercapacitors,^31^ batteries,^32^ and organic electrochemical transistors.^33^, ^34^, ^35^ PEDOT:PSS is the most notable example of polymeric MIEC, and when combined with cellulose, it forms ultrathin to ultrathick layers (10 nm to 10 µm)^36^ with record‐high combined ionic and electronic conductivities.^31^ To the best of our knowledge, there are no reports of MIEC aerogels.

**Figure 1:**
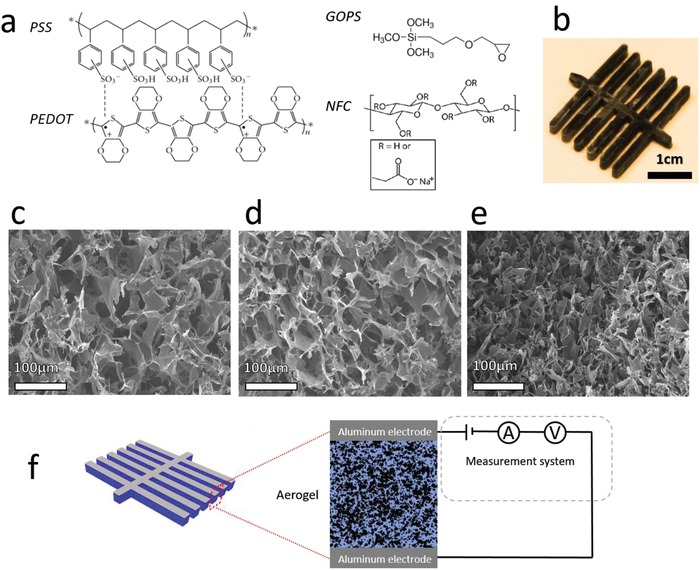
a) Chemical structures of PEDOT:PSS, GOPS, and NFC. b) Photograph of a prepared MIEC aerogel for a *P*–*T*–*H* sensor. c–e) SEM images of the aerogel under different pressures. c) 0 Pa, d) 300 Pa, and e) 600 Pa. f) Schematic diagram of a *P*–*T*–*H* sensor setup.

Here, we fabricate aerogels of polymeric MIECs and demonstrate that these materials exhibit decoupled sensitivity to pressure, temperature gradient, and humidity. When included in a device configuration, the pressure is read out as a resistance change (linear slope), the temperature is read as a steady thermovoltage (voltage threshold), and the humidity is read as a thermovoltage peak (dynamics). The resulting multiparameter *P–T–H* sensor can thus measure three physical parameters without any major cross‐talk. The material used in this work is prepared by freeze drying a water dispersion including four organic components (**Figure** **1**a): the electrically conducting polymer PEDOT provides the electronic thermovoltage, the ionic conducting polymer PSS provides the ionic thermovoltage peak, the mechanically strong nanofibrillated cellulose (NFC) forms the mechanical structure of the aerogel, and the crosslinking agent glycidoxypropyl trimethoxysilane (GOPS)^17^ introduces elasticity to the aerogel.

### 2 Results and Discussion

Previously, we have identified that the reading of the thermovoltage versus time allows us to distinguish between three classes of materials: majority electron conductors, majority ion conductors, and mixed electron–ion conductors.^29^ PEDOT:PSS is an example of polymeric MIECs^36^ where both electrons and ions have been reported to thermodiffuse efficiently.^29^ The evolution of the thermovoltage versus time in a MIEC displays a specific voltage peak that is related to thermodiffusion of ions, a phenomenon that strongly depends on humidity. After an extended period of time, the peak vanishes, and the thermovoltage level is then solely related to the electronic Seebeck effect, which typically does not depend on humidity. The introduction of the time domain, i.e., dynamics, in the measurement allows us to separate between the electronic and ionic contributions to the resulting thermovoltage. Hence, by knowing the ionic Seebeck coefficient for various humidity levels and the electronic Seebeck coefficient, the temperature gradient and humidity can exclusively be extracted, respectively.

To simultaneously measure temperature and pressure, we utilize a strategy that was previously presented and that is based on an organic thermoelectric aerogel. The thermoelectric aerogel can be optimized to display pressure‐dependent resistance and to possess a Seebeck coefficient, which is independent on pressure and temperature.^17^ By recording the steady‐state *I–V* characteristics of the material, we find that the slope is pressure dependent and the shift in the intercept of the voltage axis is directly dependent on the thermovoltage level. Hence, if the Seebeck coefficient and the resistance‐pressure characteristic are known, the *I–V* of the thermoelectric aerogel provides both pressure (change in linear slope) and temperature (shift in voltage offset).

To fabricate the thermoelectric aerogels, we blend all components (Figure 1a) into a water solution/emulsion to achieve a one‐pot synthesis: (i) PEDOT:PSS that ensures electrical conductivity and electronic Seebeck coefficient; (ii) NFC that provides mechanical strength; and (iii) GOPS that provides water stability^37^ and elasticity.^17^ We fabricated *P–T–H*‐sensing aerogels by freeze drying the water emulsion with its three components.^17^, ^25^ GOPS includes an epoxy group, which is reactive with the hydroxyl groups of the PSS and enables then crosslinking of the PEDOT:PSS material system.^37^ In addition, GOPS is expected to crosslink the NFC component, which also includes hydroxyl groups. To ensure fast response to a swift variation in humidity, the aerogel was designed into a branch‐like device structure (see Figure 1b). This will increase the surface area over volume ratio and decreases also the total diffusion length of water vapor within the aerogel. During the freeze‐drying process, the water phase slowly sublimes from its ice crystalline state, thus generating a microstructured network of voids that is gradually replaced by air, resulting in a porous and elastic aerogel. Scanning electron microscopy (SEM) analysis of the free‐standing aerogel reveals clearly the particular microstructure of the solid system (Figure 1c). Without pressure, the pore size is typically about 50–200 µm, but when a pressure is applied to the aerogel, the pore size reduces to about 30–100 µm at 300 Pa (Figure 1d) and to about 10–50 µm at 600 Pa (Figure 1e). To measure the electrical signals from the aerogel, two aluminum electrodes having the same branch‐like structure as the aerogel were prepared (see Figure 1f). The device fabrication was completed by laminating these two electrodes on the top and along the bottom of the free‐standing aerogel to achieve a sandwich device structure. The electrodes were then connected with the measurement system so that the current, as well as the voltage data, can be measured.

**Figure 2:**
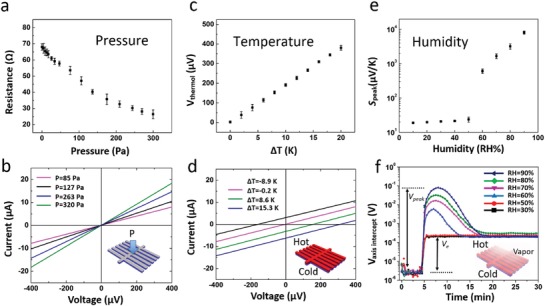
a,b) Pressure sensing. a) The resistance of the sensor has a negative correlation with mechanical pressure. The error bars are standard deviations obtained from at least five independent measurements. b) *I–V* curves measured under various pressures possess different slopes. The inserted sketch illustrates the aerogel under applied pressure. c,d) Temperature sensing, measured under RH = 30%. c) Thermoelectric voltage exhibits a linear positive correlation with the temperature gradient. The error bars are standard deviations obtained from at least five independent measurements. d) *I–V* curves measured under various temperature differences display different voltage axis intercepts. The inserted sketch illustrates the aerogel submitted to a temperature gradient, oriented in the normal direction of the device structure. e,f) Humidity sensing. e) Seebeck coefficient has a positive correlation with humidity, particularly when the relative humidity is changed between 50% and 90%. The error bars are standard deviations obtained from at least five independent measurements. f) Voltage axis intercept of *I–V* curves as a function of time, under different humidity. 10 K of temperature gradient was loaded at 4 min. The insert sketch illustrates the aerogel in a humid environment and submitted to an applied temperature gradient.

First, we investigated the pressure‐dependent performance of the *P–T–H* sensor. Without any mechanical stress or temperature gradient, and with constant humidity, the *P–T–H* sensor has a constant resistance and provides no thermovoltage, thus the current measured through the sensor has a linear response to the applied voltage and is zero at zero voltage. When a pressure is applied to the *P–T–H* sensor, the acting force causes the elastic aerogel to change in thickness and volume, which results in a change in the resistance of the conducting film as a function of the applied pressure. As shown in **Figure** **2**a, the resistance of the sensor changes with the applied pressure from 68 Ω when no pressure is applied to 26 Ω at 300 Pa. Note, the resistance value at each applied pressure can be simply calculated from the slopes of the *I–V* curves (see Figure 2b). Alternatively, tracking the pressure is simply obtained by applying a constant voltage and simultaneously reading out the current value. Notably, the device response to pressure is constant over 300 loading/unloading cycles, demonstrating the high stability of our device (Figure S1, Supporting Information). The mechanical stability could be improved even further by optimizing the elastic properties of the aerogel.

According to the typical thermoelectric mechanism, the generated electronic thermovoltage (*V*_e_) of a material is defined as: *V*_e_ = *S*_e_ × Δ*T*, where *S*_e_ is the electronic Seebeck coefficient and Δ*T* is the temperature gradient applied across the bulk of the material. When one end of our *P–T–H* sensor is in direct contact with an object, which has a different temperature than the sensor (substrate), the temperature difference between the volume of the sensor and the object can be measured by utilizing the thermoelectric effect.^38^ This temperature sensing approach is preferably performed at low humidity environments where RH ≤ 50%. Such temperature measuring experiment was carried out at a humidity of RH = 30% (see Figure 2c). The thermoelectric voltage *V*_e_ varies linearly with the temperature gradient, from 0 µV for Δ*T* = 0 K to about 400 µV for Δ*T* = 20 K. Importantly, the Seebeck coefficient changes negligibly with a temperature near room temperature.^39^ Thus, with *S*_e_ known, the Δ*T* can be easily calculated from the measured thermoelectric voltage. It should be noticed that the thermoelectric voltage can simply be read out from the voltage axis intercept of the *I–V* curves (Figure 2d). The *I–V* curves were measured for different temperature gradients: Δ*T* = −8.9, −0.2, 8.6, and 15.3 K. Importantly, the resistance of the sensor (i.e., the slope of the *I–V* curve) does not change with temperature. We achieved this by exposing the *P–T–H* sensor aerogel to the vapors of dimethylsulfoxide (DMSO), which is known to act as a secondary dopant for PEDOT:PSS,^17^ and to favor a temperature‐independent charge transport typical of a conducting system residing at the insulator‐to‐metal transition.^17^ Hence, thanks to the temperature‐independent resistance of our *P–T–H* sensor aerogel, we can decouple *P* and *T* sensing, without any cross‐talk, by applying different electric probing protocols.

At high humidity conditions (RH ≥ 60%), however, the generated thermovoltage of the *P–T–H* sensor does not only include *V*_e_ but also an ionic contribution, namely, the ionic thermovoltage *V*_i_.^29^ The data reported in Figure 2e clearly show that when RH is higher than 50%, the total Seebeck coefficient increases exponentially with humidity. Note that a similar but less obvious trend is observed for PEDOT:PSS thin films.^29^ We interpreted this in terms of PEDOT:PSS hydration state and ion mobility. When the humidity is below 50%, water content in PEDOT:PSS might not be enough for ions to move. When the humidity increases above 60%, an unobstructed pathway is established and ions start to move, contributing to the total thermovoltage. The Seebeck coefficient peak value (*S*_peak_) includes the sum of the electronic and the ionic Seebeck coefficient contributions, which becomes clear in the evolution of the thermovoltage versus time (Figure 2f), for RH > 50%. A Δ*T* = 10 K was added at *t* = 4 min. For low humidity environments (RH = 30% and 50%), the thermovoltage is exclusively generated by the electrons. As the humidity increases above 50%, however, the thermovoltage output is generated also by the ions immediately after a Δ*T* > 0 K is applied. The observed peak evolution of the thermovoltage originates from thermodiffusion of ions within the PEDOT:PSS phase^29^ and its magnitude can be directly connected to the environmental humidity. After ≈20 min, the peak is passed and the thermovoltage levels out at a constant value that corresponds to the electronic thermovoltage. Thus, the ionic thermovoltage can be calculated from *V*_i_ = *V*_peak_ − *V*_e_, where *V*_peak_ is the voltage peak value and *V*_e_ is the constant value for *t* > 20 min. The ionic contribution to the Seebeck coefficient is then $S_{i}=\frac{V_{i}}{\Delta T}=\frac{V_{\mathrm{peak}}-S_{e}\times \;\Delta T}{\Delta T}$. The humidity value can then be calculated from data in Figure 2e with *S*_i_ given from the latter equation. This means that Δ*T* must be quantified before measuring the humidity. Note that the data reported in Figure 2e have been recorded with the humidity going from 90% to 10%. A similar trend is observed when the humidity is increased from 10% to 90%, showing that the ionic thermovoltage peak is not sensitive to the sequence of the tested humidity. We have demonstrated that the MIEC aerogel‐based sensor enables an independent and exclusive measurement of the three parameters, *P*, *T*, and *H*, but one at a time by reading three independent electrical signals. We now continue reporting the investigation how to read out two sensor parameters, simultaneously.

**Figure 3:**
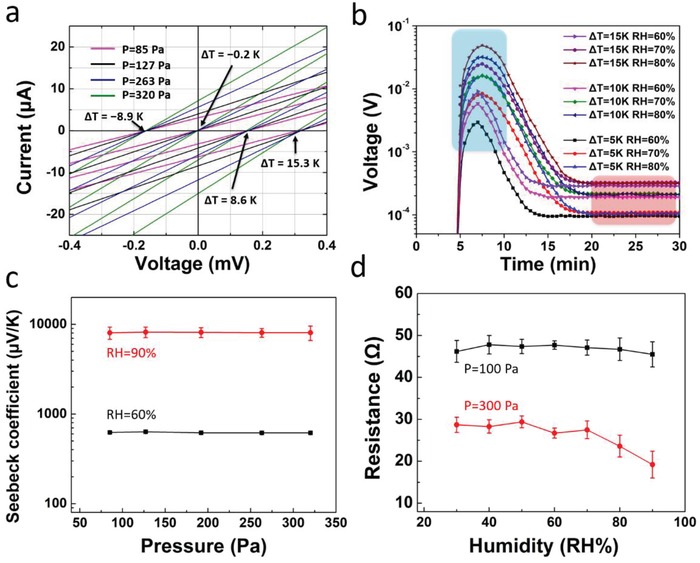
a) Measured *I–V* curves with different temperatures and pressures. The temperature of one Peltier element was constantly kept at 22 °C while varying the other one. b) Voltage axis intercept of *I–V* curves (thermal voltage output) as a function of time, under different humidity and temperatures. c) The peak Seebeck coefficient as a function of mechanical pressure, measured under RH = 90% and RH = 60%. d) The resistance of *P*–*T*–*H* sensor as a function of humidity, measured under *P* = 100 Pa and *P* = 300 Pa. The error bars are standard deviations obtained from at least five independent measurements.

For a multiparameter sensor, each parameter should ideally be quantified without any cross‐talk with respect to the others. Therefore, we studied the sensing interaction between pressure–temperature, humidity–temperature, and pressure–humidity, respectively. **Figure** **3**a displays the response of the *P–T–H* sensor upon subjecting the aerogel simultaneously to changes in pressure and temperature. When the aerogel is subject to pressure, its resistance decreases, as revealed by an increase in the slope of the linear *I–V* curves with increasing the pressure (85, 127, 263, and 320 Pa). The temperature difference between the two electrodes was controlled with two Peltier elements. A temperature gradient is then introduced, and a corresponding thermovoltage, equal to *S*_e_Δ*T*, is generated, which then shifts the intercept of *I–V* curves (Δ*T* = −8.9, −0.2, 8.6, and 15.3 K). All measurements were carried out at an RH = 50%. Hence, the *P–T–H* sensor allows for decoupling and simultaneous sensing both pressure and temperature by reading the slopes and voltage axis intercept of the *I–V* curves, respectively.

We then continue to investigate simultaneous and orthogonal sensing of temperature and humidity. Figure 3b shows different thermovoltage output curves versus time under different RH (60%, 70%, and 80%) for different Δ*T* (5, 10, and 15 K). According to the fundamental thermoelectric mechanism, Δ*T* corresponds to the electronic thermovoltage *V*_e_ achieved from the flat voltage regime established after 20 min (marked with a red foreground in Figure 3b). Note that the electronic thermovoltage depends negligibly on RH (Figure S2, Supporting Information) so that Δ*T* is extracted from the measured *V*_e_ values (and the known value of *S*_e_) without any cross‐talk with respect to variation in RH. The peak value of the voltage output (marked with a blue foreground in Figure 3b) depends on both humidity and Δ*T*. The ionic contribution to the Seebeck coefficient *S*_i_ can be calculated from $S_{i}=\frac{V_{\mathrm{peak}}-S_{e}\times \;\Delta T}{\Delta T}$ and correlated to the humidity level through the known evolution of *S*_i_ versus RH (plotted in Figure 2e). Hence, the *P–T–H* sensor allows to quantify temperature and humidity by a simultaneous decoupling of *V*_peak_ and *V*_e_ read‐out, respectively.

As a next step, we studied the effect of pressure and humidity on the Seebeck coefficient *S*_i_, in order to assess whether undesired interference or coupling affects the simultaneous sensing of these two parameters (Figure 3c). From 85 to 320 Pa, *S*_i_ is more or less constant with pressure, but varies significantly for different relative humidity levels, as illustrated by measurements performed at RH = 60% (*S*_i_ = 620 µV K^−1^) and RH = 90% (*S*_i_ = 8100 µV K^−1^). We then concluded that applying and measuring pressure for values under 300 Pa does not interfere with a simultaneous changing and measuring of the humidity, at least within the RH window of 60%–90%. We also studied the impact of humidity on the resistance values measured upon applying two different pressures. The applied pressure causes a decrease of thickness, and thus resistance. A constant relationship between pressure and thickness is necessary for an accurate measurement of pressure. However, a change in RH is expected to have an impact on the mechanical strength and/or volume of the hygroscopic aerogel. At low applied pressure (*P* = 100 Pa), the resistance of the sensor is however constant with humidity (Figure 3d). However, at high pressures (i.e., at *P* = 300 Pa) and at high RH values, a drop in resistance versus humidity is in fact measured, in this case from 28 Ω (50% RH) to 19 Ω (90% RH). We tentatively ascribed this observation to a decrease of the aerogel elasticity at higher relative humidity conditions. To verify this, the mechanical properties of aerogels were measured under different humidity conditions (Figure S3, Supporting Information). After ten compression cycles, the aerogels at high humidity environment (RH > 70%) did not recover to their original size. This indicates that there is a window of pressure and humidity where the cross‐talk between these two parameters is negligible, but it becomes significant at *P* ≥ 300 Pa and RH ≥ 70%.

**Figure 4:**
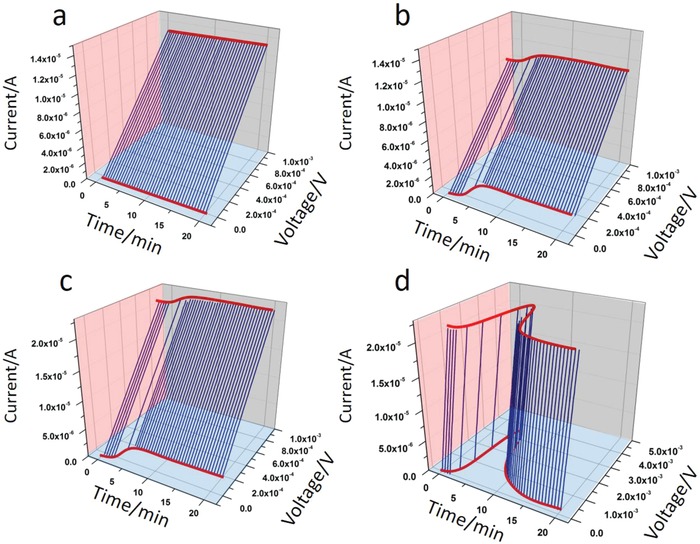
a) Measured *I–V* curves under an initial state of *P* = 85 Pa, ∆*T* = 0 K, RH = 30%. b,d) A ∆*T* = 10 K was applied at 90 s after starting the measurements. *I–V* curves measured at b) *P* = 85 Pa, RH = 30%, c) *P* = 127 Pa, RH = 30%, and d) *P* = 127 Pa, RH = 60%.

We now turn to the conceptual proof of quantifying the three environmental parameters *P*, *T*, and *H*, simultaneously and orthogonally, through the reading of the three measurable parameters “time, voltage, and current”. For this task, we consider four different probing protocols. In the initial protocol, pressure (*P* = 85 Pa), temperature (Δ*T* = 0 K), and humidity (RH = 30%) are kept constant and the *I–V* curves recorded as a function of time (**Figure** **4**a). Without any temperature gradient and under the same pressure and humidity conditions, the slopes (meaning the *I–V* plane, marked with a pink background in Figure 4a) and voltage axis intercept (meaning the voltage–time plane, marked with a blue background in Figure 4) of *I–V* curves remain constant and stable over time (0–20 min). In the second probing protocol, mechanical pressure and relative humidity were both kept constant (*P* = 85 Pa, RH = 30%). A temperature gradient of Δ*T* = 10 K was applied 90 s after initiating the measurement (Figure 4b). The *I–V* curves then start to shift along the voltage axis due to the thermoelectric effect. Within the voltage–time plane, we find that the thermovoltage increases to about 0.2 mV and then becomes constant after additional ≈60 s of heating, which is in agreement with the dynamics data of the Seebeck coefficient at RH = 30% (see Figure 2c). Figure 4c shows the *I–V* curves versus time of the *P–T–H* sensor probed and characterized in the third probing protocol. First, we applied an extra pressure of 127 Pa to the sample. Then, after 90 s, a 10 K temperature gradient is applied. Because of the same temperature difference (Δ*T* = 10 K), the *I–V* curves in Figure 4c have the same position along the time–voltage plane as the curves given in Figure 4b. Since a higher pressure is applied when probing, according to the third protocol, higher *I–V* curve slopes are generated (compare the slopes in Figure 4c with those given in Figure 4b). In the fourth probing protocol, the extra pressure of 127 Pa and a relatively higher humidity environment of RH = 60% were applied before running the measurement. A temperature gradient of 10 K was applied 90 s after that the measurement was started. In agreement with the behavior of the third probing protocol, the *I–V* curves start moving to higher voltage position after 90 s (see Figure 4d). The difference of this protocol, as compared to the third probing protocol, is that the high humidity condition activates the phenomenon of ionic thermodiffusion. The latter gives then rise to the formation of a thermovoltage peak (as also found in the data given in Figure 2e,f for RH = 60%). Thus, from Figure 4d, we can conclude that we can quantify, orthogonally, the values of applied “pressure, temperature, and humidity” from the slopes, the voltage position of the flat area (reached after about 15 min of heating), and the peak value of the thermal voltage within the voltage–time plane, respectively.

### 3 Conclusion

In summary, we have explored a polymer MIEC aerogel, based on PEDOT:PSS and nanofibrillated cellulose, in a multiparameter sensor device to enable independent measurement of *P*, *T*, and *H*. This is achieved by using a transduction protocol of the sensor device characteristics into a current–voltage–time coordinate system. The transduction mechanisms are based on the mechanoresistive effect, electronic Seebeck, and ionic Seebeck effects. The peculiar evolution of the thermovoltage versus time allows us to distinguish between ionic and electronic Seebeck effects. From those, the temperature gradient and relative humidity can be calculated in a straightforward manner. Reading *P*, *T*, and *H* in a truly decoupled fashion is demonstrated for the first time with one single material, and it is achieved in a very simple two‐terminal device. Further development of this technology will include material optimization to extend the dynamic range windows for each targeted parameter, without any cross‐talking. Further, we will also consider extending the ensemble into four, perhaps even five, orthogonal sensing parameters, since considerably more than three static and/or dynamic device curve features, coupled to exclusive device mechanisms, can be represented in one simple device configuration. More research is also needed to understand, in depth, the interplay between the various physicochemical phenomena in these aerogels. Efforts will be devoted to develop easy manufacturing protocols, targeting printing techniques, to enable easy integration of *P–T–H* sensors in IoT labels also including application‐specific integrated Si‐chips. The goal is then to demonstrate that a two‐terminal multiparameter sensor can be utilized to considerably reduce the number of contact pads on the silicon chip, for a multifunctional IoT label.

As an additional pathway, new manufacturing methods and techniques, such as screen printing, will be explored to enable local deposition of small aerogel sensor pixels, arranged into a sensor matrix. This would then generate multiparameter sensing in each cross‐point of a passively or actively addressed electronic matrix system. Our hope is that this blazes the trail toward 2D mapping of temperature, pressure, and humidity and would thus go far beyond the state of the art in electronic skins either for medical applications or robotics.

## 4 Experimental Section

*P–T–H Sensor Fabrication*: A *P–T–H* sensor was fabricated based on an organic thermoelectric aerogel. This so‐called MIEC (PEDOT:PSS‐NFC‐GOPS) aerogel was prepared by PEDOT:PSS (Heraeus Clevios, PH1000, 1.3 wt% of PEDOT:PSS), NFC (carboxymethylated, 0.5 wt%), and GOPS (Alfa Aesar, 97 wt%). These three components were mixed as a solid ratio of 1:1:0.2 in solution form by an ULTRA‐TURRAX T‐10disperser. This mixed solution was then dropped into a branch‐like lab‐made aluminum mold and covered with a glass slide. The solution with the aluminum mold was then frozen by liquid nitrogen, followed by freeze drying (Benchtop Pro, SP SCIENTIFIC) under −60 °C and 50 µbar for 12 h. As a result, water molecules were removed and replaced with air. The branch‐like structure causes a higher surface area of the aerogel and allows it to absorb more water molecules when detecting humidity. The density of the aerogel was about 0.0108 mg mm^−3^. The MIEC aerogel after freeze drying was then put into an oven under 140 °C for 30 min to crosslink GOPS with PEDOT:PSS and NFC. To increase the pressure sensitivity and remove the cross‐talk between pressure sensing and temperature sensing, the crosslinked aerogel was then treated by DMSO vapor. For DMSO treatment, a glass Petri dish was placed on top of a hotplate (set temperature at 60 °C) with the MIEC aerogel inside. A few drops of DMSO were placed around the sample but not in contact with it. A DMSO‐treated branch‐like MIEC aerogel was connected with two aluminum electrodes on the bottom and top. The MIEC aerogels, as well as the electrodes, were then sandwiched between two Peltier elements on bottom and top to control temperature.

*Characterizations*: Flexible carbon fibers were used to connect the device to the measurement system (Keithley 4200) to apply voltage and measure *I–V* curves. Different pressures were applied by loading different balance weight on top of the top Peltier element. Environmental temperature control and Seebeck coefficient measurements were implemented use lab‐made thermoelectric setup. Humidity environment was applied by a lab‐made humidity chamber that could provide humidity of RH = 10% to RH = 100%. The data reported in Figure 2e have been measured with the humidity going from 90% to 10%.

## Conflict of Interest

The authors declare no conflict of interest.

## Supporting information

Supplementary
