# Supplementary material for: A Multiparameter Pressure–Temperature–Humidity Sensor Based on Mixed Ionic–Electronic Cellulose Aerogels
Source: Adv Sci (Weinh). 2019 Feb 7;6(8):1802128. doi: 10.1002/advs.201802128 (PMC6468975; doi:10.1002/advs.201802128)
Supplement: Supplementary file 1 — Supplementary [file ADVS-6-1802128-s001.pdf]

Copyright WILEY-VCH Verlag GmbH & Co. KGaA, 69469 Weinheim, Germany, 2019.

Supporting Information

for *Adv. Sci.,* DOI: 10.1002/advs.201802128

A Multiparameter Pressure–Temperature–Humidity Sensor Based on Mixed Ionic–Electronic Cellulose Aerogels

*Shaobo Han, Naveed Ul Hassan Alvi, Lars Granlöf, Hjalmar Granberg, Magnus Berggren, Simone Fabiano, and Xavier Crispin**

Supporting Information

A Multi-Parameter Pressure-Temperature-Humidity Sensor Based on Mixed Ionic-Electronic Cellulose Aerogels

Shaobo Han, Naveed Ul Hassan Alvi, Lars Granlöf, Hjalmar Granberg, Magnus Berggren Simone Fabiano, Xavier Crispin*

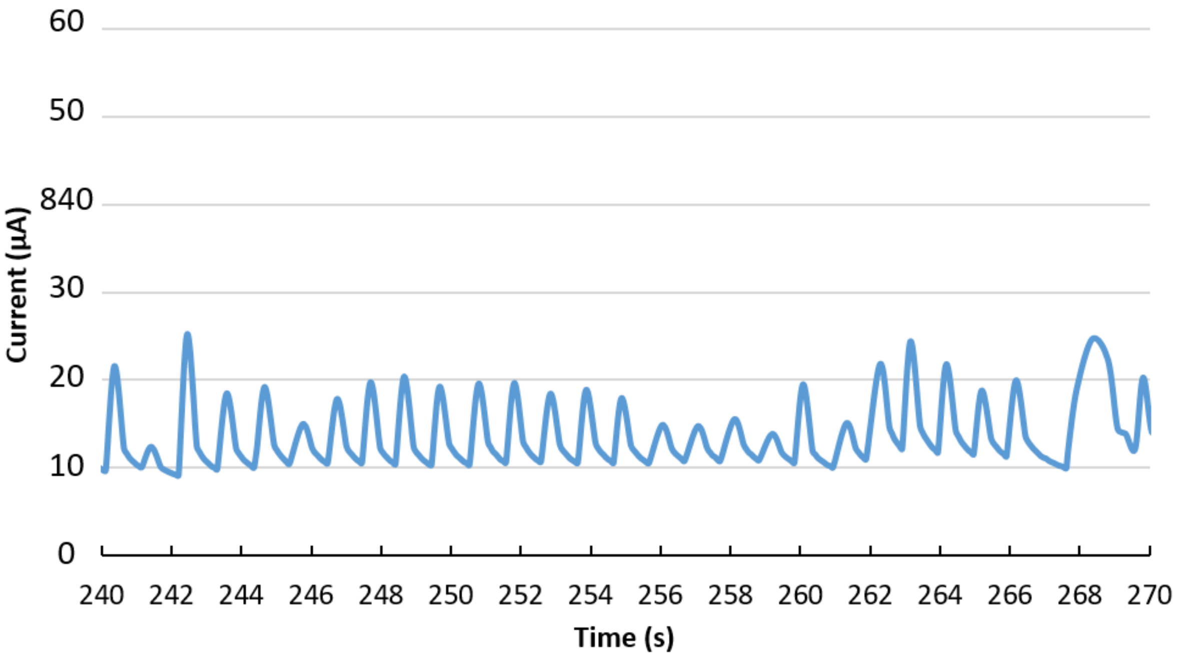

Figure S1 The current response (at a constant voltage of 1 mV) along time with intermittent finger touch. The value of current was increased by finger press and recovered to the original value in about one second.

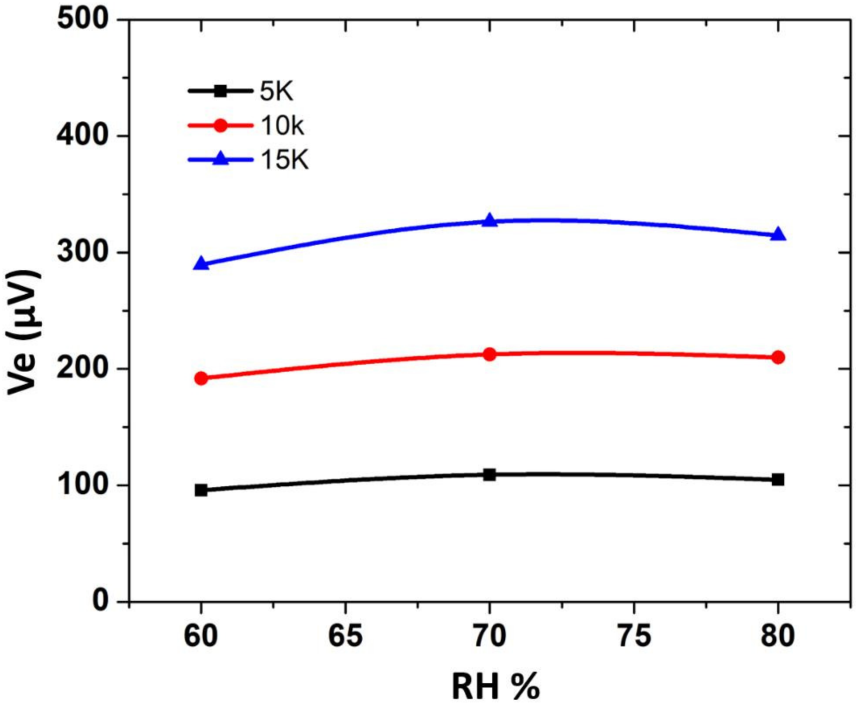

Figure S2 Electronic thermovoltage as function of relative humidity, under different temperature difference.

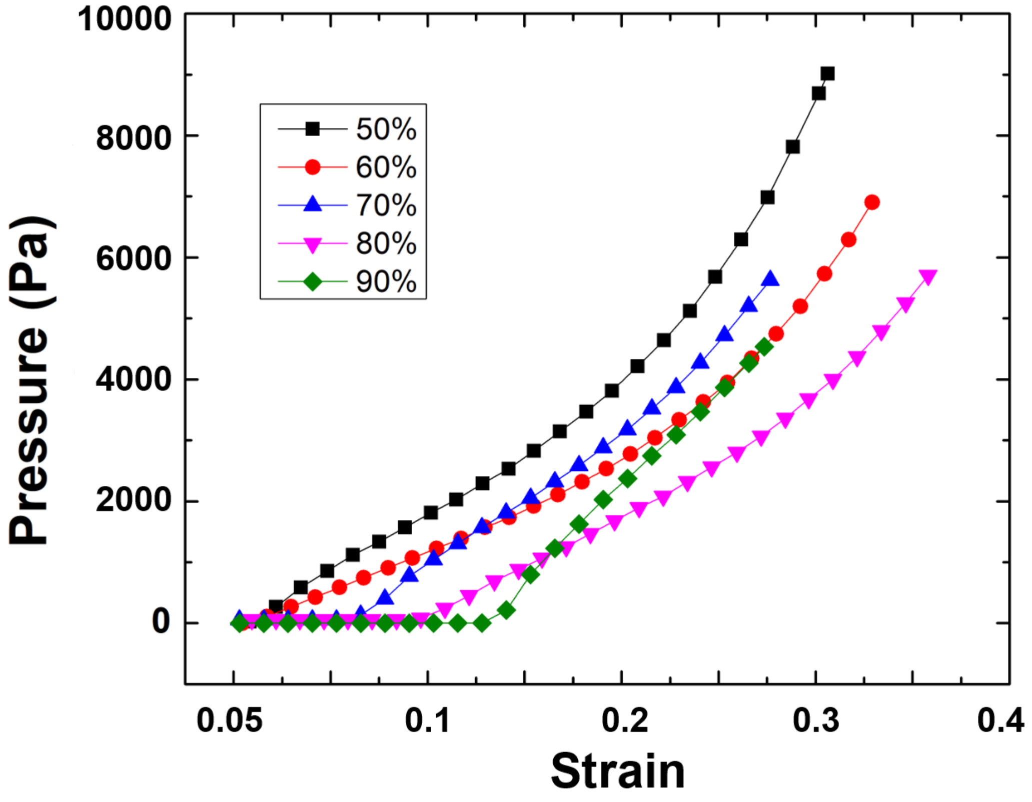

Figure S3 Pressure applied on an aerogel as function of strain, after 10 time compress. Different data were measured under different humidity, as the insert table showing.
